# RNAbrowse: RNA-Seq De Novo Assembly Results Browser

**DOI:** 10.1371/journal.pone.0096821

**Published:** 2014-05-13

**Authors:** Jérôme Mariette, Céline Noirot, Ibounyamine Nabihoudine, Philippe Bardou, Claire Hoede, Anis Djari, Cédric Cabau, Christophe Klopp

**Affiliations:** 1 Plate-forme bio-informatique Genotoul/Biométrie et Intelligence Artificielle, INRA, Castanet-Tolosan, France; 2 Plate-forme SIGENAE/Génétique Cellulaire, INRA, Castanet-Tolosan, France; Georgia Institute of Technology, United States of America

## Abstract

Transcriptome analysis based on a de novo assembly of next generation RNA sequences is now performed routinely in many laboratories. The generated results, including contig sequences, quantification figures, functional annotations and variation discovery outputs are usually bulky and quite diverse. This article presents a user oriented storage and visualisation environment permitting to explore the data in a top-down manner, going from general graphical views to all possible details. The software package is based on biomart, easy to install and populate with local data. The software package is available under the GNU General Public License (GPL) at http://bioinfo.genotoul.fr/RNAbrowse.

## Introduction

The massive sequencing cost decrease has attracted a large community of new users, some of them studying organisms for which the reference genome sequence is still not available. When trying to understand mechanisms taking place at the gene level they usually would start with a de novo transcriptome assembly approach. Software packages such as Trinity [1] or Oases [2] are mature enough to produce reliable contigs from short reads. The analyses performed on the assembled contigs generate a large amount of heterogeneous results including variations, functional annotation and expression measurements. The processing steps of these pipelines are usually shared inside the community but the parameters, the tools and the reference databases used are specific. The results are often provided throught a WEB server including a BLAST query form and download links.

Cbrowse [3] is a WEB environment presenting this kind of results throught graphical views and query forms. The functional annotation part is not implemented yet and the query possibilities are very limited. The Galaxy [4] engine provides users with an interface to create and track workflow executions. It already embeds RNA-Seq analysis and assembly components. However, none of them offers user-friendly query and vizualisation features designed for RNA-Seq de novo annotation.

In its last version (0.8), biomart [5] is developed as an easily extensible query infrastructures which can be specialized in the presentation of focused data types. On top of the database and beside the proposed query forms it is possible to add new pages as plug-ins in order to present data in a user-friendly way.

## Results

RNAbrowse permits sequencing facilities and, even small, bioinformatic teams to give a user-friendly access to RNA-Seq de novo results, helping biologists to analyse and extract meaningful information from their data.

RNAbrowse includes two components: a web-based user interface and an administration command line tool presented here-after.

### Implementation and installation

RNAbrowse is based on a newly defined biomart schema specialized in RNA-Seq de novo assembly. It includes two marts, storing contig and variant data, and some extra tables for meta-data. The WEB-based user interface is implemented as a biomart plugin using the latest jquery and highcharts features. To perform all third parts requests, the software takes advantage of the biomart API and implements several biomart processors to prepare the data before presenting it.

The command line administration tool is based on a workflow execution environment called jflow. It is developed in Python. The installation requires a standard unix server and a MySQL database. The downloadable installation archive, provided on the project web-site, has only to be uncompressed before setting up a first instance. In order to ease the testing, a minimal and a complete example dataset are included in the archive.

### WEB interface

The web interface was designed to display the data starting with general views before zooming into detailed ones. It is organised in four levels. The first, called the instance level, corresponds to the home page of the web-site presenting all the available projects. A project gathers the data of an assembly. The second level is entered after having chosen a project. The introduction page contains a picture of the assembled species, a description text and a table including all the analysis processing steps with software names, parameters and versions in order to ease materials and methods writing.

**Figure 1:**
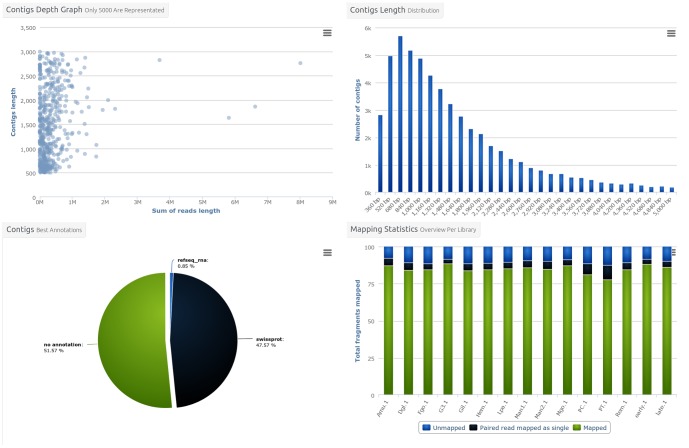
Contigs overview figures. From the main contig page, graphics synthesizing informations on the contig set can be displayed. The presented graphs are contigs depth versus size plot (top-left), contigs length distribution (top-right), contig best annotations pie chart (bottom-left) and libraries mapping bar chart (bottom-right). All graphics can be printed or downloaded in four different formats (PNG, JPEG, PDF, SVG).

**Figure 2:**
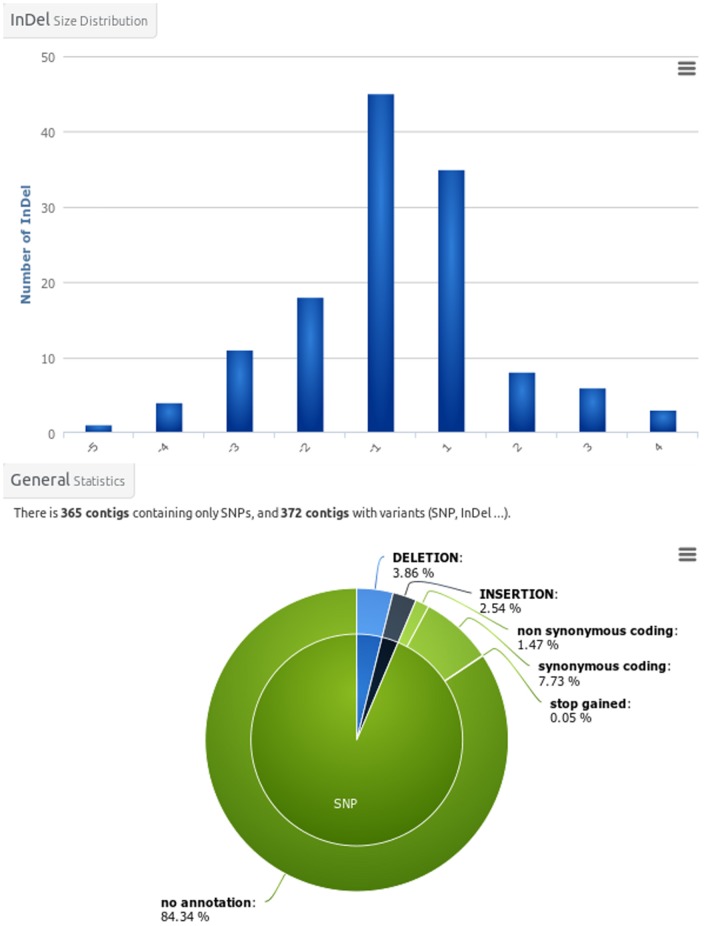
Variants overview figures. From the main variant page, graphics synthesizing informations on the variant set can be displayed. The presented graphs are InDels size distribution (top) and Indels annotations pie chart (bottom).

**Figure 3:**
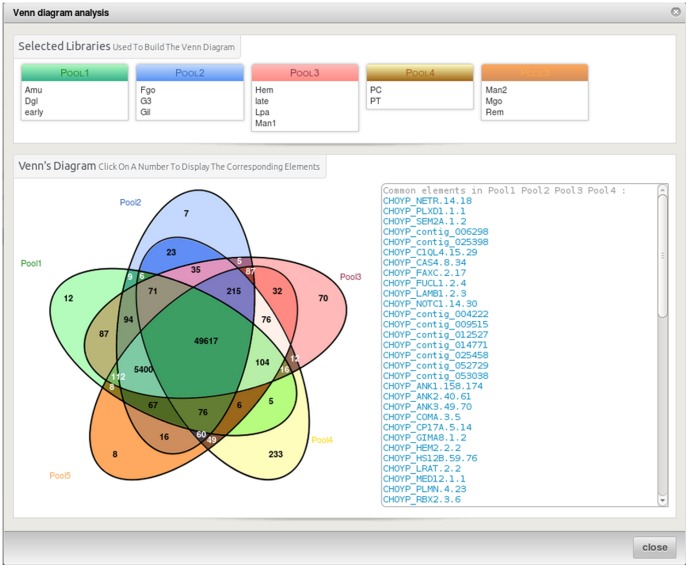
The Venn diagram shows the number of contigs shared between libraries and the specific ones. It has been built using libraries alignment data. To build a new diagram the user has to select the libraries he wants to have in each pool (from two to five). If the user clicks on a figure in the graph the list of corresponding contigs will appear in the list box on the right hand side. Clicking on the contig names redirects to the corresponding page.

The third level is accessed through the menu bar items at the top of the project page and presents general informations about contigs and variants. It also includes the download page. The contigs (Figure 1) and variants (Figure 2) overview pages include a set of graphics showing general statistics, containing for example the contig length histogram, alignment rate bar-chart, mostly represented species in the functional annotation histogram or variant types distribution as a pie-chart. These graphs enable rapid assembly validation and comparison. For contigs, the next section includes detailed information about the different sequenced libraries and provides access to tools such as Venn diagrams (Figure 3) and a differential digital display. The last section corresponds to the favourite contigs or variants table which can be updated by the user while exploring detailed views. The forth level sections gathers all information on a contig or a variant and provides multiple tools to analyse them further.

**Figure 4:**
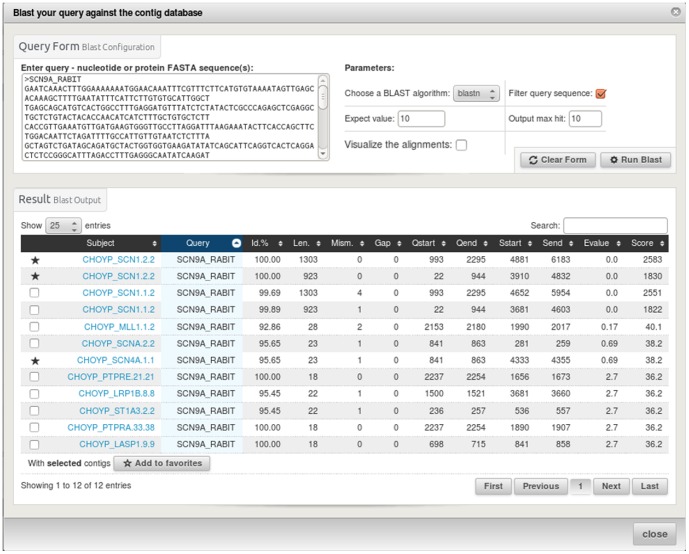
The blast query form allows to search the contigs as a database using as input sequence(s) in Fasta format. Others parameters which can be used in this query are blast program (blastn, blastx), expected value and maximum number of outputs to be shown. The blast results are shown in a table allowing to add new contigs as favorite. Ticking the alignment checkbox enables to browse the alignment results.

**Figure 5:**
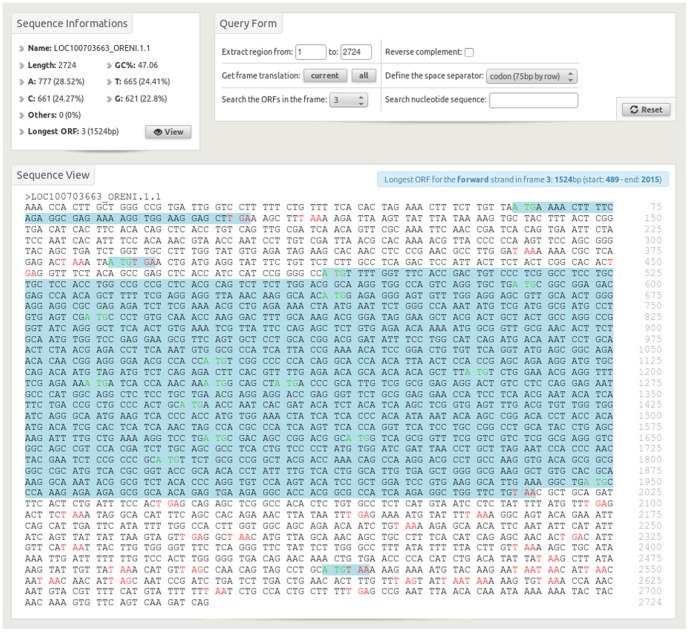
The sequence view provides informations such as nucleotide content and longest open reading frame (ORF). The possible starts are presented in green, stops in red and ORFs in blue. The query form permits different actions on the sequence such as extraction, reverse complementation, translation in different frames, ORF presentation and text search.

**Figure 6:**
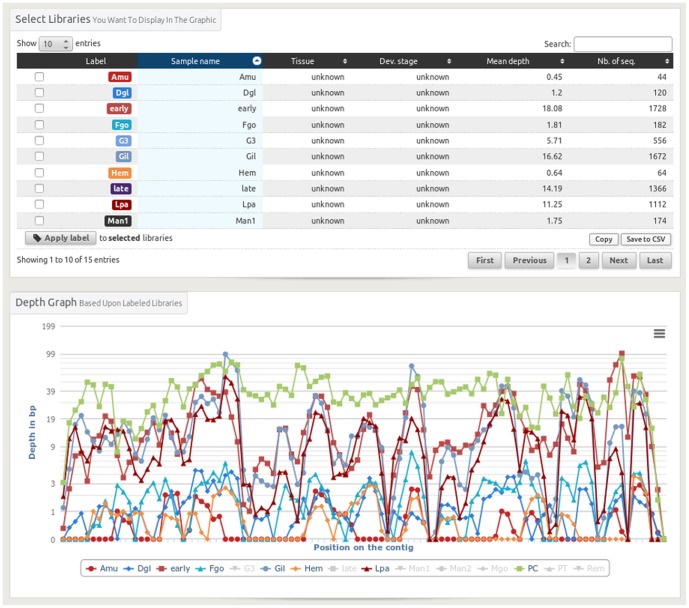
The contig depth view enables to visualise the coverage of the reads of the different libraries on a given contig. Each library has a defined colour in the table and the same one on the graphic. It is possible to modify the graphical layout by averaging different library depths. This is typically to be used when you work with replicates.

To illustrate these features we could imagine a simple use case in which a user would like to find all contigs corresponding to a gene for which the sequence is available for another species. This can be done by an alignment versus the contigs using the blast query form (Figure 4) or by a name or description search using the biomart form. The user can then add the found contigs to the favourite table. For each contig, the sequence can be extracted to perform a multiple alignment in order to check if different splice forms have been assembled. All possible open reading frames can be sought (Figure 5). Annotations can be graphically displayed in jbrowse [6]. It is also possible to graphically verify if the expression levels along the contig are conserved between libraries (Figure 6).

All generated graphics can directly be printed from the web-page or downloaded in JPG, PNG, PDF, SVG formats or as a CSV file. Tables can be sorted on all columns, text searched and exported to the clipboard or as CSV files. The download page files are organised as topics. It is possible to download a single file, all the files of a topic or to get the text file containing all the URLs of a topic in order to download the files from the command line. The Download section gathers raw files but also processed ones, enabling users to perform further analyses.

A demo site containing a small set of results is accessible at: http://ngspipelines.toulouse.inra.fr:9012/.

### Administration tools and extensions

The administration interface is a command line tool with which the environment can be set up. It includes database creation, data formatting and loading and web server managing. The loading process requiers standard file formats, provided by most commonly used tools. During the database upload phase, datafile format compliance is checked. It is possible to set up a minimum environment with just three files: a contig Fasta file, the corresponding annotation file and an alignment file. The reads quantifications can be provided as an input file or calculated during the upload process. The number of input files being potentially quite important for large projects, a configuration file template is provided. It can be used as a loading script parameter.

Depending on the provided inputs, RNAbrowse processing steps can be quite time consuming. Thus, in a production environment, the tool can be set up to use different schedulers such as condor, sge, moab, workqueue or mpi-queuein in order to parallelize the loading. If the loading process fails, a recovery command is available to rerun it.

The website provided to the biologists can be secured using the biomart Open-ID or jetty realm features.

Users with some programming knowledge can add new graphics by loading the corresponding data in the analysis table and writing a javascript module.

## Discussion

RNAbrowse is a simple and efficient solution to give access to RNA-Seq de novo results on the Internet. It includes many features that help biologists to analyse and extract biologically meaningful information from their data. The installation and user manuals as well as a general documentation are available on the project website: http://bioinfo.genotoul.fr/RNAbrowse. The software has been designed to be easily extended by developpers having some biomart insights.
