# Correction: RNAbrowse: RNA-Seq De Novo Assembly Results Browser

**DOI:** 10.1371/journal.pone.0106187

**Published:** 2014-08-19

**Authors:** The PLOS ONE Staff

The following information is missing from the Funding section: This work on RNAbrowse has been partially supported by the BLINDTEST ANR grants (Agence Nationale pour la Recherche), which paid for the publication fees.
